# Elastin-like Recombinamer Hydrogels as Platforms for Breast Cancer Modeling

**DOI:** 10.1021/acs.biomac.2c01080

**Published:** 2023-01-04

**Authors:** Barbara Blanco-Fernandez, Arturo Ibañez-Fonseca, Doriana Orbanic, Celia Ximenes-Carballo, Soledad Perez-Amodio, Jose Carlos Rodríguez-Cabello, Elisabeth Engel

**Affiliations:** † Institute for Bioengineering of Catalonia (IBEC), The Barcelona Institute of Science and Technology (BIST), Baldiri Reixac 10-12, Barcelona 08028, Spain; ‡ CIBER en Bioingeniería, Biomateriales y Nanomedicina, CIBER-BBN, Madrid 28029, Spain; § BIOFORGE Lab, CIBER-BBN, University of Valladolid, Paseo de Belén 19, 47011 Valladolid, Spain; ∥ IMEM-BRT Group, Department of Materials Science and Engineering, EEBE, Technical University of Catalonia (UPC), Barcelona 08019, Spain

## Abstract

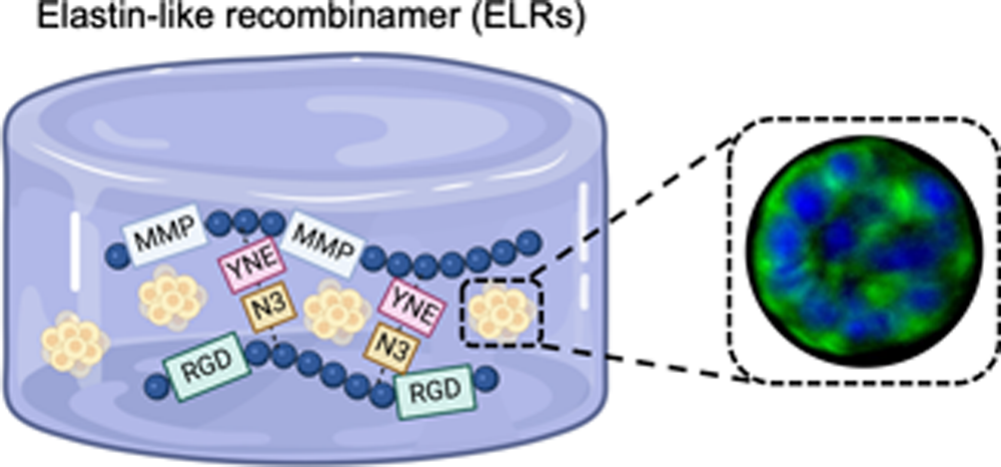

The involvement of the extracellular matrix (ECM) in tumor progression has motivated the development of biomaterials mimicking the tumor ECM to develop more predictive cancer models. Particularly, polypeptides based on elastin could be an interesting approach to mimic the ECM due to their tunable properties. Here, we demonstrated that elastin-like recombinamer (ELR) hydrogels can be suitable biomaterials to develop breast cancer models. This hydrogel was formed by two ELR polypeptides, one containing sequences biodegradable by matrix metalloproteinase and cyclooctyne and the other carrying arginylglycylaspartic acid and azide groups to allow cell adhesion, biodegradability, and suitable stiffness through “click-chemistry” cross-linking. Our findings show that breast cancer or nontumorigenic breast cells showed high viability and cell proliferation for up to 7 days. MCF7 and MCF10A formed spheroids whereas MDA-MB-231 formed cell networks, with the expression of ECM and high drug resistance in all cases, evidencing that ELR hydrogels are a promising biomaterial for breast cancer modeling.

## 1 Introduction

Cancer is one of the main causes of death worldwide. Indeed, pharmaceutical companies are investing significant economic resources in oncology R&D. However, most new candidates fail in clinical trials due to efficacy and off-target effect problems, despite positive results in preclinical trials.^1,2^ Undoubtedly, one of the main reasons for this low success in clinical trials is associated with the lack of effective preclinical models which closely recapitulate the tumor complexity.^3^ Therefore, the development of more biomimetic tumor models could help in the discovery of new treatments as well as lead to a deeper understanding of cancer physiopathology.^4^ Tumors are composed of cancer cells, stromal cells, secreted factors, and the extracellular matrix (ECM), known as the tumor microenvironment (TME), all of these being components involved in the tumor progression and drug outcome.^5−13^ The ECM is the main structural element of the TME (constituting up to 60% of the TME),^14,15^ and it also provides biochemical and biomechanical cues involved in cellular functions and tumor progression.^16−18^ The ECM is formed by a complex 3D nanofibrous network of proteins, polysaccharides, glycoproteins, and proteoglycans.^15,18−21^ During tumor progression, there is an alteration in the ECM remodeling that provokes a stiffening of the tissue,^22,23^ a progression and invasiveness of the tumor,^24^ or a drug response,^25^ among others. The active role of the ECM in tumor progression has evidenced the necessity to recreate the tumor ECM to develop more clinically translatable preclinical models. Currently, 2D and animal models are the most used preclinical models in cancer research,^26,27^ even though these models cannot fully mimic the key hallmarks of human tumors. Indeed, cells growing in 2D cannot recreate the TME and differ in their biological properties from cancer cells in the *in vivo* scenario,^28−32^ displaying a less malignant phenotype.^33^ Furthermore, anatomical and physiological divergences between species^34^ or the lack of an immune system^35^ in animal models impacts the translatability between preclinical and clinical trials.^34,36^ Consequently, the development of 3D *in vitro* cancer models is gaining interest in order to overcome these limitations. Cells growing in a 3D environment can recapitulate many key features of tumors such as cancer cell morphology and gene and protein expression, hypoxia, or drug response.^37−44^ As a result, they have proved to be physiologically relevant and reproducible platforms for cancer research.^45^ Two main approaches have been followed in 3D models, scaffold-free and scaffold-based systems. In scaffold-free platforms, cancer cells grow to form cell clusters or spheroids, which can recapitulate many key features of tumors.^46−48^ Nevertheless, the role of the ECM in tumor progression and drug response^8,20,49−52^ has motivated the development of tumor ECM-like biomaterials to develop scaffold-based systems where cells are anchored or encapsulated in them.^53,54^ Traditionally, these platforms have been formulated as hydrogels fabricated from proteins, polysaccharides, synthetic biomaterials, native ECM, or their combinations.^45,55,56^ Recently, hydrogels made of self-assembling peptides have emerged as suitable platforms for cancer modeling due to the easiness to tailor their mechanical and biochemical properties to recapitulate the tumor ECM, and the similarities between the nanofiber network and the ECM architecture.^57−59^ To date, several peptides have been tested in the development of cancer models, including EAK16, RADA16, Fmoc-FF, Fmoc carrying (RGD), Peptigel, and h9e, for investigating cancer cell behavior and drug response.^45,60^

In this work, we aimed to test whether elastin-like recombinamers (ELRs) could be suitable biomaterials to mimic the breast tumor ECM. ELRs are fabricated through recombinant technology to mimic elastin, an ECM component, and are formed by repetitions of the sequence VPGXG (V being valine, P being proline, G being glycine, and X being any amino acid except l-proline). These polypeptides self-assemble above certain temperatures due to hydrophobic interactions,^61^ and their sequences can be genetically engineered to incorporate specific amino acids or bioactive sequences,^62^ such as cell adhesion motifs like the peptide RGD or protease-degradable sequences.^63,64^ In addition, lysine’s amine group can be grafted with chemical groups such as azide or cyclooctyne groups to form chemical hydrogels through click-chemistry.^65^ ELR hydrogels have already evidenced their versatility and capacity to recreate the ECM in several biomedical applications,^63,64,66,67^ but to the best of our knowledge, ELR hydrogels have not been evaluated to develop tumor models. Here, we describe for the first time the use of an ELR hydrogel cross-linked through click chemistry for the development of breast cancer models. The hydrogel is formed by two ELRs, one including RGD to promote cell adhesion and another incorporating protease cleavage sites that can be biodegraded by matrix metalloproteases (MMPs), which are overexpressed in breast tumors. We anticipate that ELR hydrogels will possess great potential for the study of cancer physiopathology and drug discovery.

## 2 Experimental Methods

### 2.1 Materials and Reagents

Acti-stain 488 phalloidin was purchased from Cytoskeleton Inc. Advanced Dulbecco’s modified Eagle’s medium (aDMEM), Dulbecco’s modified Eagle’s medium–Nutrient Mixture F-12 l-glutamine (DMEM/F12), Dulbecco’s phosphate-buffered saline (DPBS) 10×, glutamine, horse serum, penicillin–streptomycin, phosphate-buffered saline (PBS), propidium iodide (PI), and Vybrant DiO cell-labeling solution were acquired from Thermo Fisher Scientific. Anti-Rabbit IgG (H+L), CF 647 antibody produced in goat (SAB4600184), bovine serum albumin (BSA), calcein AM, cholera toxin, doxorubicin, epidermal growth factor (EGF), fetal bovine serum (FBS), hydrocortisone, insulin from bovine pancreas, paraformaldehyde (PFA), Triton X-100, and 4′,6-diamidino-2-phenylindole (DAPI) were purchased from Sigma-Aldrich. Anti-collagen I antibody (ab34710), anti-collagen III antibody (ab7778), anti-collagen IV antibody (ab6586), anti-fibronectin antibody (ab2413), and goat serum were purchased from Abcam. Collagen type I (Col1) was isolated from rat tail tendons.^68^ The Col1 content was measured by microBCA (Thermo Fisher Scientific) as previously described.^56^

### 2.2 ELR Synthesis and Chemical Functionalization

ELRs were synthesized by recombinant DNA technology.^69^ ELR carrying RGD (HRGD6) and ELR with MMP-degradable sequences (HE5) were biosynthesized as previously described.^64,70^ Briefly, the peptide gene was cloned into the plasmid vector pET-25b(+) (Novagen, Merck). Then, *Escherichia coli* [BLR(DE3) strain, Novagen, Merck] was transformed with the plasmid, and a clone expressing the ELR peptide was cultured in a bioreactor (Applikon Biotechnology B.V.). Finally, ELR was purified through inverse transition cycling and dialysis, sterilized through filtration (0.22 μm, Nalgene, Thermo Fisher Scientific), and freeze-dried. Then, the amine group of the side chain of lysine amino acids from ELR peptides was functionalized as previously described.^71^ HRGD6 was functionalized with azide groups (55–65% of grafting, HRGD6-N3), and HE5 was grafted with cyclooctyne groups (30–40% of grafting, HE5-C).^63^

### 2.3 Cell Culture

MCF7 (HTB-22, ATCC), and MDA-MB-231 (HTB26, ATCC) breast cancer cells were cultured in aDMEM supplemented with FBS (10%), glutamine (1%), and penicillin–streptomycin (1%). MCF10A cells (ATCC HTB26, ATCC) were cultured in DMEM/F12 supplemented with horse serum (5%), penicillin–streptomycin (1%), hydrocortisone (500 μg/mL), insulin (10 μg/mL), choleric toxin (100 ng/mL), and EGF (20 ng/mL).

### 2.4 Cell-Laden Hydrogel Fabrication

HRGD6-N3 and HE5-C were dissolved in both cell media at 4 °C at a concentration of 35.71 and 128.56 mg/mL, respectively. When cells were above 70% confluent, cells were harvested and suspended in the respective cell media at 4 × 10^6^ cells/mL. Cells and polymer solutions were kept at 4 °C, and 96-well plates and pipette tips used for the manipulation of the solutions were stored at −20 °C to avoid protein precipitation during its manipulation. For the preparation of 1 mL of the ELR pregel, 250 μL of cells was mixed with 250 μL of HE5-C and then mixed with 500 μL of HRGD6-N3, which gives hydrogels of 50 mg/mL ELR and a molar ratio of HE5-C/HRGD6-N3 of 1.8:1, which has been previously established as suitable for hydrogel fabrication.^63,64^ Then, this pregel was vortexed and incubated at 4 °C for 8 min. 10–100 μL of pregels was added to 96-well plates and then incubated at 37 °C for 15 min. Afterward, cell media was added on top of the hydrogel. The cell concentration in the hydrogel was 10^6^ cells/mL. Cell-laden hydrogels were kept in a humidified incubator (37 °C, 5% CO_2_), and cell media was changed every day.

### 2.5 Cell Viability in Cell-Laden Hydrogels

Cell viability was measured using a live/dead staining on days 1, 3, 7, 10, and 14 of hydrogels of 50 μL (*N* = 3). Hydrogels were harvested and washed with DPBS. Then, they were incubated with a solution of Calcein AM (2 μM) and PI (4 μM) in DPBS (20 min, 37 °C) to stain viable and dying cells, respectively. Afterward, the hydrogels were washed with DPBS, and *Z*-stack images were acquired with a confocal laser scanning microscope (Leica TCS-SP5, Leica Microsystems). The cell viability was determined with FIJI software.^72^

### 2.6 Cell Proliferation in Cell-Laden Hydrogels

Cell proliferation was measured with alamarBlue cell viability assay reagent (AB, Thermo Scientific) on days 1, 3, 5, 7, 10, and 14 on hydrogels of 10 μL (*N* = 4). At each time point, cell media was replaced by 150 μL of 10% AB solution in cell media and incubated for 2 h. The AB fluorescence intensity was quantified using a plate reader (excitation 560 nm; emission 590 nm). In order to compare the cell proliferation, collagen type I (Col1) hydrogels at 4 mg/mL were used as controls. Cell number was calculated using the corresponding calibration curve (MCF10A, 10^3^ to 5 × 10^4^; MCF7, 2.5 × 10^3^ to 2 × 10^5^; MDA-MB-231, 5 × 10^3^ to 7.5 × 10^4^; *r*^2^ > 0.99).

### 2.7 Immunofluorescence

Cell morphology within the hydrogels was observed by actin/nuclei staining (*N* = 3). After 1, 3, 7, and 14 days in culture, hydrogels were rinsed with DPBS twice, fixed with 4% PFA in PBS [10 min, room temperature (RT), 10 rpm], and washed again with PBS (3 × 3 min, RT, 10 rpm). Then, samples were permeabilized with 0.05% Triton X-100 in PBS (5 min, RT, 10 rpm), and washed again with PBS (3 × 3 min, RT, 10 rpm). Afterward, the cytoskeleton and nuclei were stained with phalloidin (100 nM, 45 min, RT, 10 rpm) and DAPI (10 min, RT), respectively. *Z*-stacks were acquired with a confocal laser scanning microscope (Leica TCS-SP5, Leica Microsystems) and a Thunder Imager 3D live cell microscope (Leica Microsystems). The area of the spheroids formed by the cells was determined with FIJI software, by manually quantifying the area occupied by the cytoskeleton of each spheroid.^72^ The areas of a minimum of 12 spheroids per replicate were measured. The number of cells per spheroid was also manually counted, by quantifying the number of nuclei in each spheroid. A minimum of 11 spheroids per replicate were counted.

The expression of ECM proteins by cells was evaluated through immunofluorescence (*N* = 3). Cell-laden hydrogels were rinsed twice with DPBS, fixed with 4% PFA in PBS (10 min, RT, 10 rpm), and washed again with PBS (3 × 3 min, RT, 10 rpm). Then, samples were permeabilized with 0.05% Triton X-100 in PBS (5 min, RT, 10 rpm), washed again with PBS (3 × 3 min, RT, 10 rpm), and blocked with 6% BSA in PBS containing 10% goat serum (1 h, RT, 10 rpm). Afterward, cells were incubated with the primary antibody diluted at a 1:500 ratio in the blocking solution (overnight, 4 °C). Hydrogels were then washed with the blocking solution (3 × 10 min, RT, 10 rpm) and incubated with the secondary antibody diluted at 1:1000 in the blocking solution (1 h, RT, 10 rpm). Finally, a cytoskeleton/nucleus staining with phalloidin/DAPI was performed as specified before. *Z*-stacks were acquired with a Thunder Imager 3D live cell microscope (Leica Microsystems).

### 2.8 3D Invasion and Migration in ELR Hydrogels

To study the invasiveness of breast cancer cells (BCCs) into the ELR hydrogel, MDA-MB-231 or MCF7 cells were cultured on top of the hydrogel, and its invasion toward the hydrogel was monitored by fluorescent microscopy (*N* = 3). Briefly, MDA-MB-231 or MCF7 cells were labeled with Vybrant DiO according to the manufacturer’s instructions. 10 μL of ELR hydrogels was prepared in a μ-Slide Angiogenesis chamber (Ibidi). Then, 50 μL of the labeled cells was added on top of the gel (100,000 cells/mL). Two cell media were used, one containing 0% FBS and one containing 10% FBS. Gels were imaged with a Thunder Imager 3D live cell microscope (Leica Microsystems) after 3 h and 1, 2, and 3 days in culture. A mosaic tile of each XY conforming the well with zetas of 10 μm of each well was acquired. To quantify the cell invasion, the volume of migration at each time was measured with Fiji software.^72^ To avoid the effect of any surface defect, the volume of the cells at time 3 h was used to normalize the values.

### 2.9 Doxorubicin Efficacy

Doxorubicin efficacy against BCCs and MCF10A cultured within ELR hydrogels was evaluated by AB. Briefly, 10 μL of cell-laden hydrogels was cultured for 7 days and treated with doxorubicin for 2 days at different concentrations (*N* = 3). Then, hydrogels were washed with PBS, and the AB was carried out as specified in section 2.6. The cellular viability was determined using nontreated hydrogels as negative controls. 2D experiments were run in parallel.

### 2.10 Statistical Analysis

All data are represented as the mean value ± standard deviation. All statistics were conducted with GraphPad Prism 8.0 software (GraphPad Software). A *t*-test analysis was carried out to study whether two groups were statistical different. Multiple groups were compared using one-way (for 1 independent variable) or two-way (for 2 independent variables) ANOVAs. Differences were considered statistically significant when *p*-values were below 0.05.

## 3 Results and Discussion

### 3.1 Cell Encapsulation and Viability in ELR Hydrogels

**Scheme 1:**
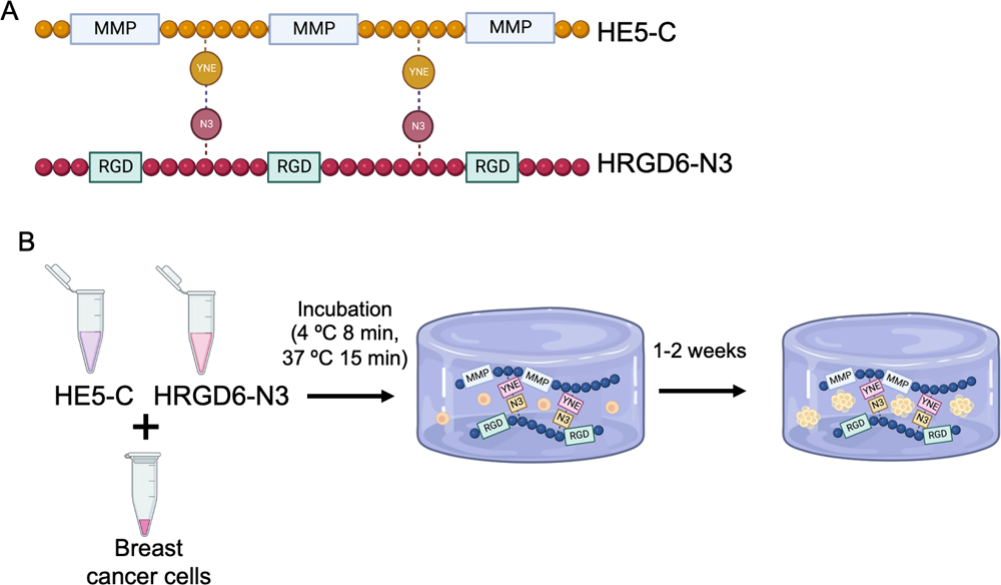
ELR Hydrogel Fabrication and Cell Encapsulation (A) Schematic representation of the ELR sequences used. (B) Protocol to produce the ELR cell-laden hydrogels. Breast cancer or nontumorigenic breast epithelial cells were mixed with HE5-C and HRGD6-N3 and incubated at 4 and at 37 °C to allow the hydrogel formation. Hydrogels were kept under culture for up to 2 weeks.

ELR hydrogels have been widely explored in the areas of tissue engineering and regenerative therapies, showing promising advances in the field.^61,63−67,70^ In this work, we explore the suitability of HE5-C/HRGD6-N3 hydrogels to develop breast cancer models. This transparent and porous hydrogel with a storage modulus of 600 Pa has been previously evaluated in tissue regeneration, evidencing its similarities with the native ECM and showing encouraging results in skeletal muscle and cardiac tissue regeneration.^63,64^ This ELR hydrogel has two different ELR polypeptides, HE5 and HRGD6 (Scheme 1A). HE5 ELR has been designed to be biodegradable by cells through enzymatic digestion. In particular, it contains sequences that can be biodegraded by MMPs (MMP-2, MMP-9, and MMP-13) and cathepsin K,^64^ which are overexpressed in breast tumors and bone metastasis, respectively.^73−76^ For its part, HRGD6 incorporates the peptide RGD to guarantee cell adhesion. HE5 and HRGD6 ELRs were synthesized through genetic engineering as described before.^63,64^ Afterward, HE5 was functionalized with an activated alkyne group (cyclooctyne), and HRGD6 was grafted with azide groups as reported before to enable the formation of hydrogels through click chemistry.^64^ Cell-laden hydrogels were later fabricated through the click reaction strain-promoted alkyne–azide cycloaddition, which has been reported to be biocompatible and occurs under physiological conditions without the requirement of a catalyst,^63,64,66,67,70^ by combining both grafted ELRs and cells. The fabrication of the cell-laden hydrogels is summarized in Scheme 1B. Three types of cells were encapsulated within the hydrogels: nontumorigenic breast epithelial cells (MCF10A) and two types of breast cancer cells (BCCs), a luminal A and nonmetastatic cell line (MCF7), and a triple-negative and metastatic cell line (MDA-MB-231).

**Figure 1:**
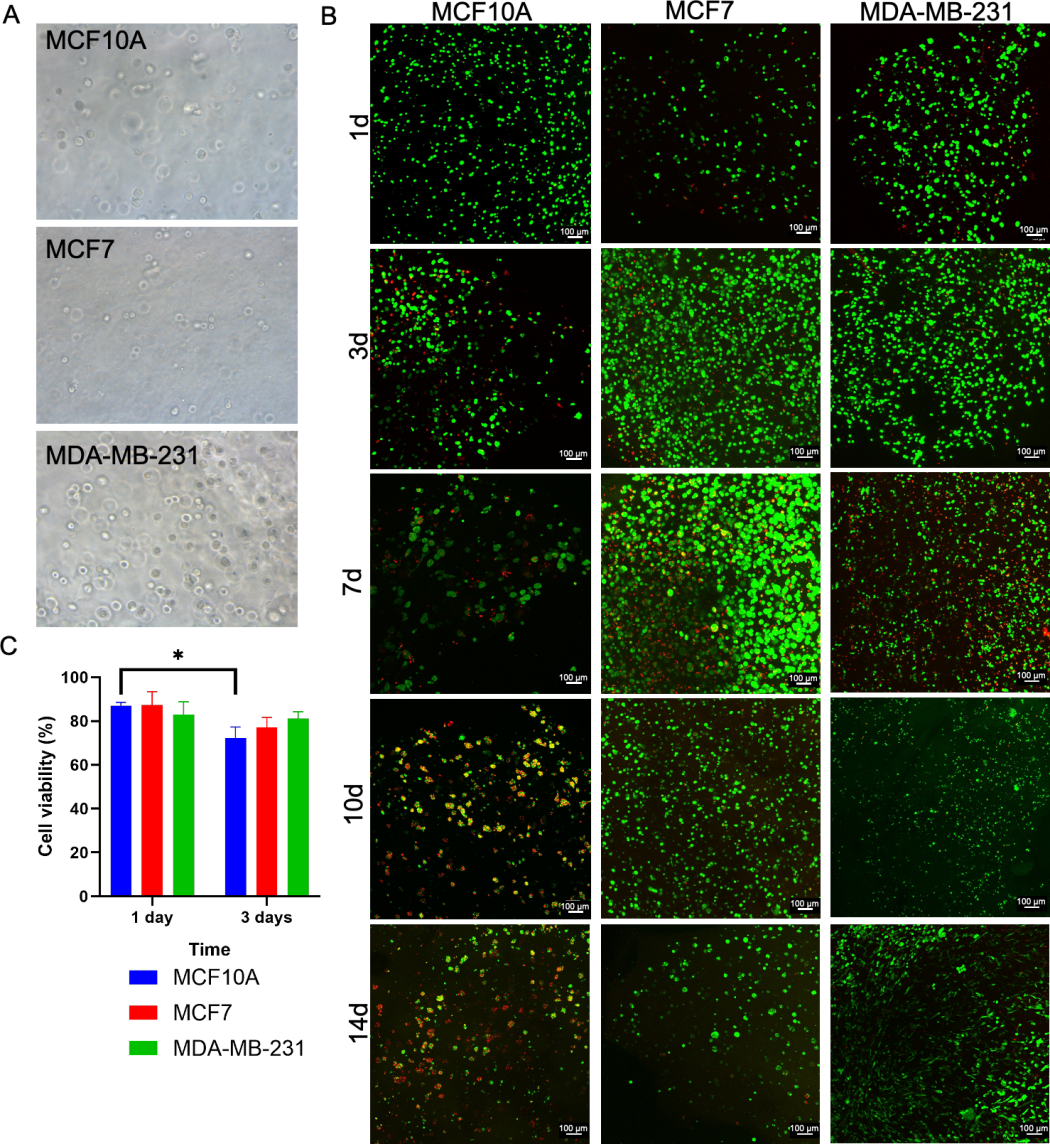
Cell encapsulation and viability in ELR hydrogels. (A) Cell-laden hydrogels under an optical microscope (DM IL LED, Leica, 20× objective) on day 1. (B) Calcein/PI staining of MCF10A, MCF7, and MDA-MB-231 within ELR hydrogels after 1, 3, 7, 10, and 14 days of incubation (green, alive cells; red, dead cells; scale bar = 100 μm). (C) Cell viability after 1 day.

Hydrogels were transparent and allowed the visualization of the cells with an optical microscope, the cells being individually distributed in the hydrogels (Figure 1A). After 1, 3, 5, 7, 10, and 14 days of incubation, the cell viability on the cell-laden hydrogels was assessed through vital staining with calcein/PI (Figure 1B,C). High cell viability was observed on day 1 and 3, being above 70% in all cell types (Figure 1C). However, a small reduction in cell viability was observed for MCF10A cells at day 3. After 1 week in culture, a high cell viability was still observed, although cell death was present to some extent. Once the spheroids were formed in the hydrogels, the high cell density per spheroid hindered the precise quantification of the cell viability. Therefore, only a qualitative assessment of the viability could be carried out. Cell death continued increasing in the case of MCF10A cells on days 10 and 14 but was not relevant in the case of BCCs. Nevertheless, ELRs were able to support the cell viability for 1 week, which has not been achieved with other peptide gels without the incorporation of tumor ECM components into the gel, such as Matrigel or laminin.^77^ These results indicate the high cytocompatibility over time of ELR hydrogels for the 3D culture of BCCs and nontumorigenic breast cells, which is compatible with breast cancer modeling.

### 3.2 Cellular Proliferation

**Figure 2:**
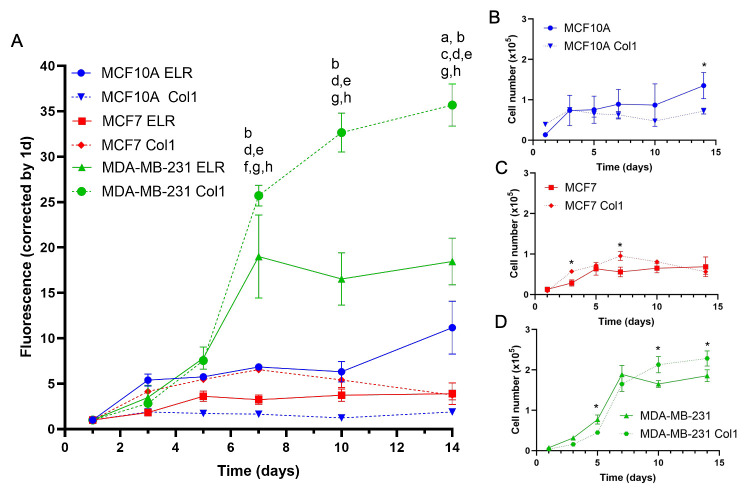
Cell proliferation in ELR hydrogels and Col1 by AB. (B) Fluorescence intensity of MCF10A, MCF7, and MDA-MB-231 within ELR and Col1 hydrogels over time normalized by the intensity of day 1: (a) *p* < 0.05 MCF10A ELR vs MCF10A Col1; (b) *p* < 0.05 MDA-MB-231 ELR vs MDA-MB-231 Col1; (c) *p* < 0.05 MCF10A ELR vs MCF7 ELR; (d) *p* < 0.05 MCF10A ELR vs MDA-MB-231 ELR; (e) *p* < 0.05 MCF7 ELR vs MDA-MB-231 ELR; (f) *p* < 0.05 MCF10A Col1 vs MCF7 Col1; (g) *p* < 0.05 MCF10A Col1 vs MDA-MB-231 Col1; and (h) *p* < 0.05 MCF7 Col1 vs MDA-MB-231 Col1. Cell number of MCF10A (B), MCF7 (C), and MDA-MB-231 (D) within ELR and Col1 hydrogels over time (*p < 0.05 ELR vs Col1).

An AB assay was used to determine the cell number and proliferation in ELR hydrogels (Figure 2). As the cell number differs from each cell type on day 1, fluorescence values were normalized by day 1 to determine if the differences among conditions were responsible for the different cell numbers across time (Figure 2A). MDA-MB-231 cells were able to proliferate to a higher extent in ELR hydrogels than MCF10A and MCF7 (Figure 2A), with an 18.5-fold increase in the cell number after 14 days in culture, whereas MCF7 showed a 3.9-fold increase, and MCF10A cells had a 11.2-fold increase. This result might be due to the higher degree of malignancy of MDA-MB-231 cells, as we could also observe this phenomenon in the Col1 controls. We found that, within our scaffolds, BCCs proliferated only up to 5–7 days, showing a constant proliferation rate until the end of the culture period. This phenomenon has been reported before using peptide hydrogels.^60,78^ On the other hand, MCF10A were able to increase their proliferation for up to 14 days despite the high mortality observed at this time point (Figure 1B).

In order to compare the cell proliferation with other biomaterials used in cancer modeling, cells growing in Col1 hydrogels were run in parallel as controls (Figure 2A–D). MCF10A cells did not show any statistically significant difference between Col1 and ELR up to 10 days in culture (Figure 2B). However, cells growing in ELR gels showed higher cell numbers than Col1 hydrogels after 10 days in culture. On the other side, MCF7 cells showed higher cellular densities in Col1 gels at shorter times than ELRs, which started to decay after 1 week (Figure 2C). Nevertheless, MCF7 cells growing in ELRs could slightly increase the cellular densities up to 14 days in culture. Despite the higher cell densities of MDA-MB-231 in ELR gels than Col1 gels at shorter times (up to 7 days), this invasive cell type did not experiment cellular proliferation after 1 week in culture whereas, in Col1, it was possible to proliferate up to 2 weeks (Figure 2D). These findings indicate that cell proliferation in ELR hydrogels is dependent on cell characteristics.

### 3.3 Cell Morphology in ELR Hydrogels

**Figure 3:**
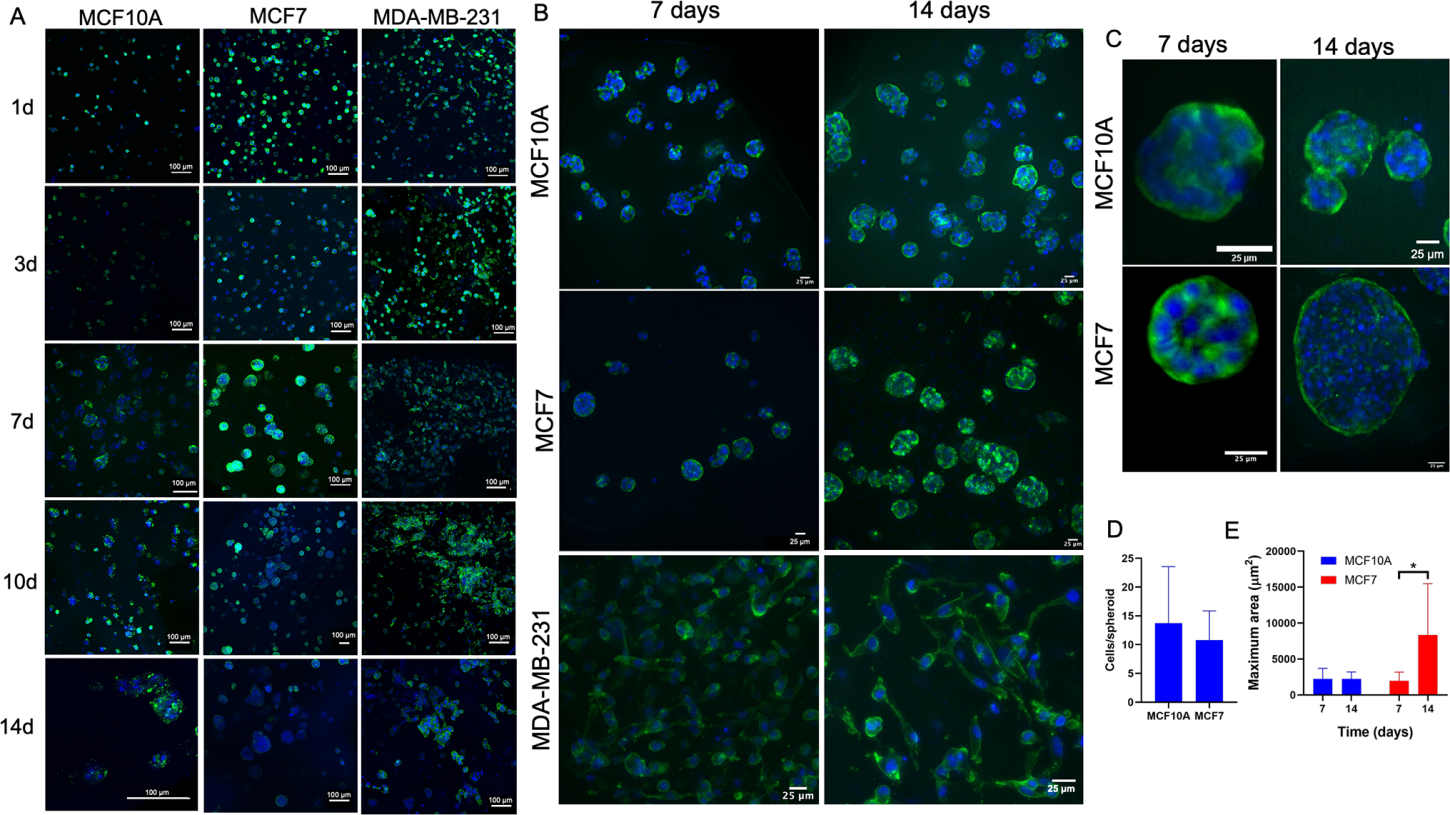
Cellular morphology in cell-laden ELR hydrogels over time. (A) Cellular morphology and spheroid formation over time in ELR hydrogels. MCF10A, MCF7, and MDA-MB-231 were stained with phalloidin-488 and DAPI after 1, 3, 7, 10, and 14 days of incubation. Images were acquired with a confocal microscope (green, cytoskeleton; blue, nuclei; scale bar = 100 μm). Cellular morphology in cell-laden ELR hydrogels after 1 week and 2 weeks in culture. (B) MCF10A, MCF7, and MDA-MB-231 cells stained with phalloidin-488 and DAPI [green: cytoskeleton; blue: nuclei; scale bar = 25 μm]. (C) Close-ups of the spheroids formed by MCF10A and MCF7 (scale bar = 25 μm). (D) Number of cells per spheroid after 1 week. (E) Area of the spheroids.

The cellular morphology within ELR hydrogels was determined through cytoskeleton/nuclei staining. All cells were individually distributed on days 1 and 3, with MCF7 and MCF10A having a more circular morphology whereas MDA-MB-231 had a more elongated shape (Figure 3A and Figures S1–S3). On day 7, MCF7 and MCF10A cells were forming spheroids and cell aggregates (Figure 3A–C). Indeed, MCF7 and MCF10A’s capabilities to form spheroids in other peptide hydrogels have already been described.^60,79,80^ High variability was observed in the cell number per spheroid in MCF10A and MCF7 at day 7, with no differences between conditions, these being values between 3 and 25 cells for MCF7 and between 6 and 30 cells for MCF10A (Figure 3D). Large variability was also seen in the spheroids area (Figure 3E). This phenomenon is expected due to the spontaneous formation of the spheroids in the hydrogel and has been already observed with other synthetic polypeptides.^60^ After 2 weeks in culture, we could observe an increase in the number of spheroids (Figure 3A–C). Nevertheless, only MCF7 spheroids showed an increase in size over time (*p* < 0.001, Figure 3E), as no differences in MCF10A spheroid size could be detected (Figure 3E). In addition, MCF10A did not form any acini on the gels over time. Nevertheless, this finding is consistent with other authors’ work that reported the requirement of the supplementation of the peptide gel matrix with Matrigel to form these structures.^77^

On the other hand, MAD-MB-231 cells showed an elongated shape, similar to the cell morphology reported in collagen matrices^81^ and peptide gels.^60,78^ Loose cell networks could be visualized in the hydrogels at day 7, and these networks continued growing and colonizing the hydrogel up to day 14 (Figure 3A,B and Figure S3), which has previously been observed in other peptide hydrogels.^60,78^

### 3.4 ECM Production

**Figure 4:**
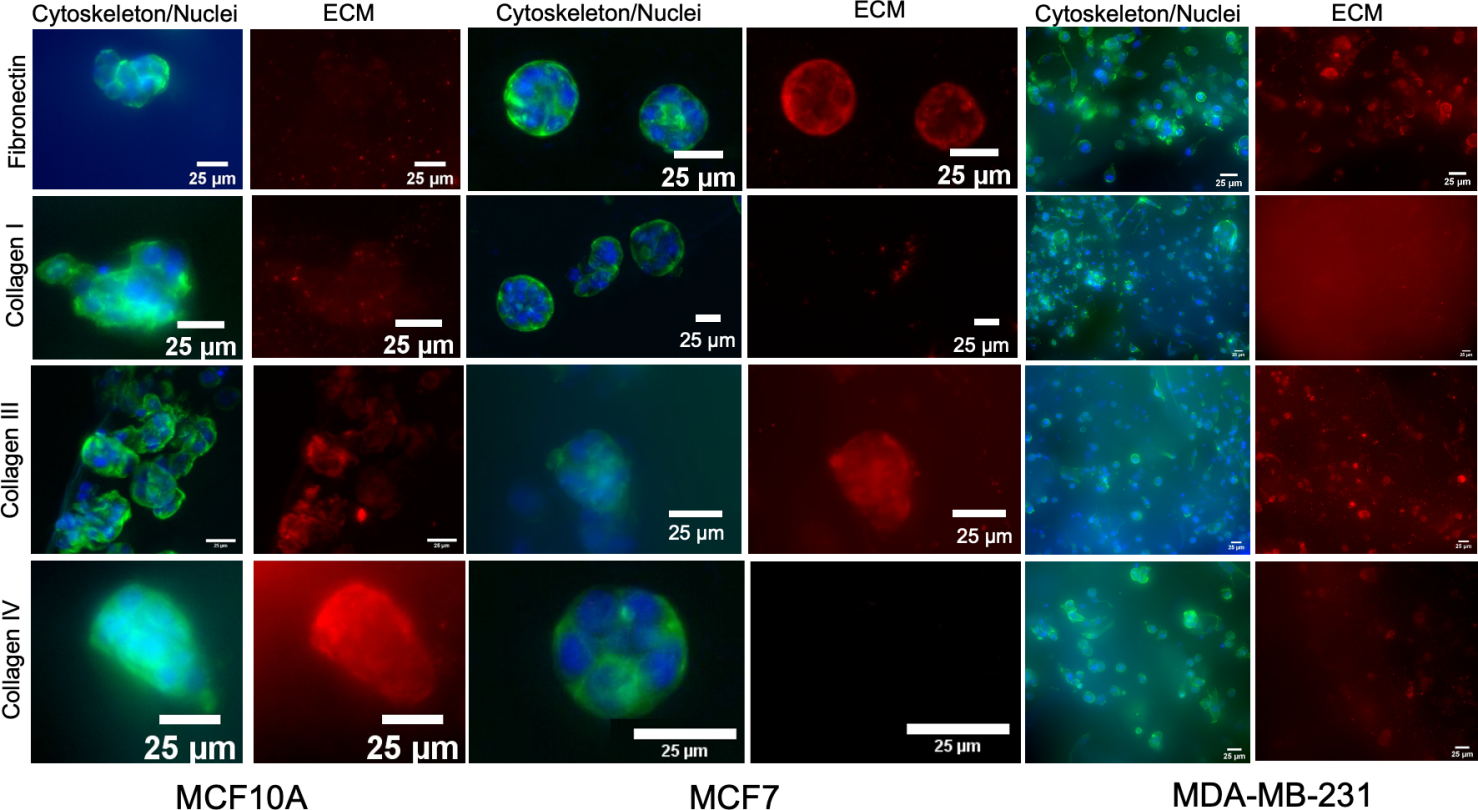
ECM production by MCF10A, MCF7, and MDA-MB-231 cells in ELR hydrogels. Fibronectin and collagen I, III, and IV expression after 7 days in culture. Close-ups of the spheroids stained with phalloidin-488, DAPI, and antibody against the ECM (green, cytoskeleton; blue, nuclei; red, ECM; scale bar = 25 μm).

During the tumor progression, BCCs and cancer-associated fibroblasts remodel the tissue ECM to support the tumor growth and invasion as well as reduce the drug response.^24,25^ Indeed, the breast tumor ECM interactions with BCCs and stromal cells play an important role in the cancer outcome.^74,82^ The synthetic origin of ELR hydrogels allows the distinction of the endogenous ECM deposition from the gel. This characteristic could allow the study of the ECM secretion by cells as well as an evaluation of the crosstalk between endogenous ECM and BCCs. Therefore, an immunofluorescence staining was carried out to evaluate if BCCs and nontumorigenic breast cells could produce ECM in ELR hydrogels, and it was possible to visualize its production *in situ* through immunofluorescence. We evaluated the secretion of four ECM proteins that are overexpressed in breast tumors (collagen types I, III, and IV as well as fibronectin) (Figure 4 and Figure S4).^82^

Immunofluorescence staining confirmed the expression of ECM proteins by BCCs and MCF10A. Spheroids formed from the nontumorigenic MCF10A cells expressed collagen type IV, probably indicating the formation of a basement membrane (Figure 4 and Figure S4).^77^ In addition, the expression of collagen type III on day 7 was observed, and fibronectin on day 14, but not collagen type I, which is consistent with the ECM composition produced in 2D cultures.^83^

On the other hand, BCCs expressed fibronectin (Figures 4 and Figure S4), which is a hallmark of breast cancer and is associated with more invasive breast tumors and linked to a poor prognosis.^82,84^ Studies in 2D have also shown the expression of collagen IV by MCF7 and MDA-MB-231,^83^ and therefore, we also assessed its expression in our models. We could only observe the expression of this protein on day 14, especially in the case of MCF7 cells. As the expression of collagen IV has been linked to the production of MMP-9 via the Src- and FAK-dependent pathway in breast cancer,^85^ the expression of this protein could be linked to a higher production of MMPs by BCCs in ELR hydrogels. Collagen III was also secreted to a great extent by BCCs, which is also overexpressed in breast tumors and linked to a bad prognosis.^82^ Surprisingly, little or no collagen I production was observed in all BCCs, which has previously been observed for MDA-MB-231 cells in peptide gels.^60^ Nevertheless, the expression of ECM proteins such as fibronectin and collagen III already indicates the production of a tumor ECM as well as the display of cell–ECM interactions within the gel.

### 3.5 BCCs’ Invasiveness on ELR Hydrogels

**Figure 5:**
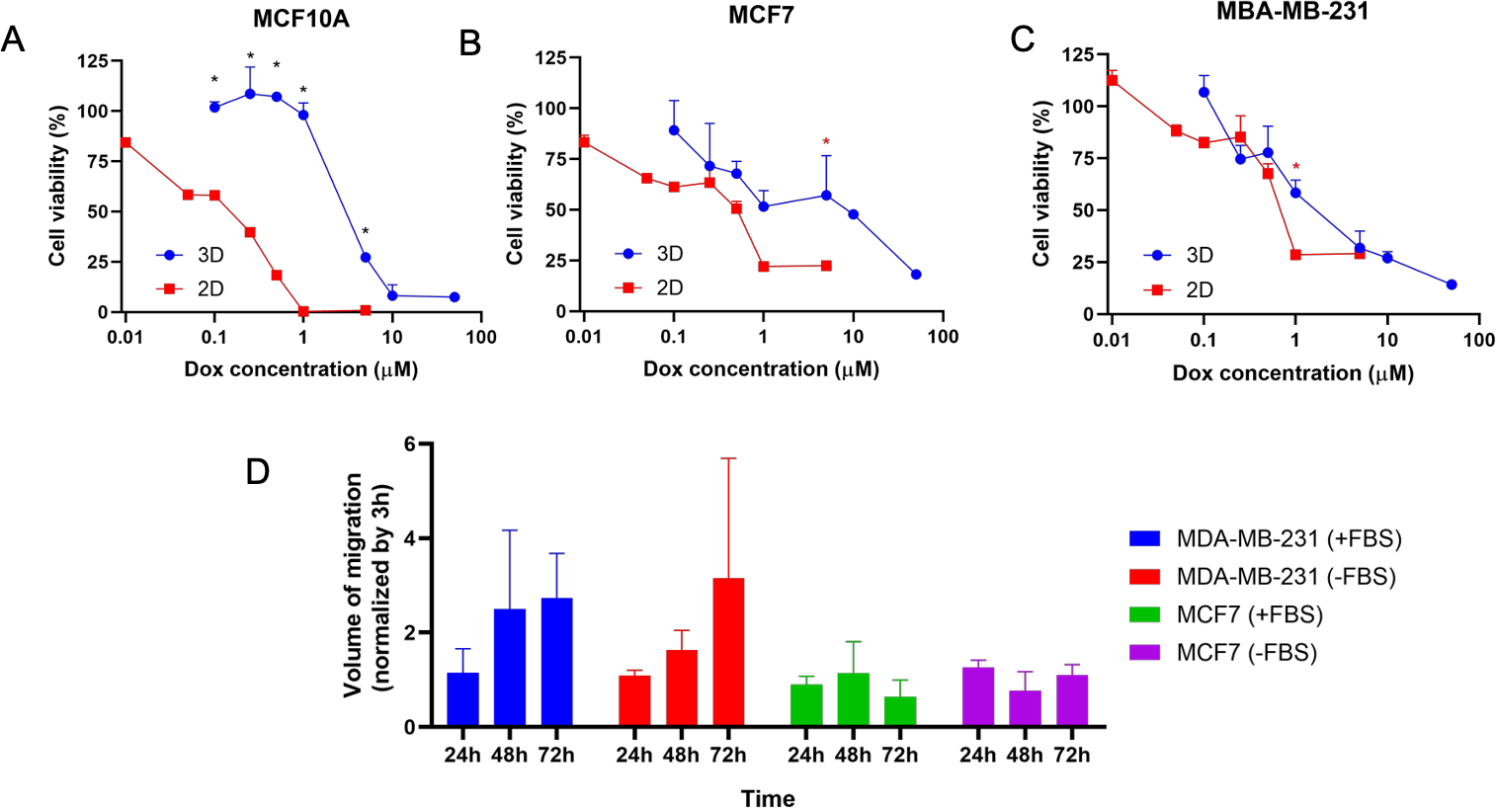
Doxorubicin efficacy and cell invasion in ELR hydrogels. Doxorubicin (Dox) efficacy against MCF10A (A), MCF7 (B), and MDA-MB-231 (C) growing in ELR hydrogels (3D) or in a monolayer (2D) after 48 h. (D) Cell invasion in ELR hydrogels of MDA-MB-231 and MCF-7 with (+FBS) and without (−FBS) the presence of FBS in the cell media. Values marked with ∗ are statistically significant (black, *p* < 0.001; red, *p* < 0.05).

With the purpose of determining whether ELR hydrogels could be suitable platforms to study cancer cell invasiveness, we assessed the ability of BCCs to invade ELR hydrogels. Poorly invasive (MCF-7) and invasive (MDA-MB-231) BCCs were seeded on top of ELR hydrogels, and the volume of gel that these cells invaded (volume of migration) was determined at different times. These values were normalized by the volume occupied by the BCCs after 3 h of the cell seeding to ensure that defects on the gel surface were not masking the results. In addition, cells seeded without FBS in the media were used as controls to determine if the invasion was promoted by cell migration or cell proliferation. MCF7 stayed on the gel surface showing no cell invasion, as values were close to 1 in all of the different time points and in the presence and absence of FBS (Figure 5D), showing that the noninvasive BCCs were not able to invade the gel in the time scale used. Despite the fact that MDA-MB-231 showed a 2-fold increase in the volume of migration after 48 h in culture, in the absence or presence of FBS, these differences were not statistically significant. However, these results show that cells can infiltrate the ELR hydrogels and migrate through them due to their invasive phenotype. We hypothesize that the migration and invasion of MDA-MB-231 is supported by the MMP-cleavable sequences in ELRs, as it has been shown that MDA-MB-231 releases MMP2 and MMP9.^86^

### 3.6 Doxorubicin Efficacy

To determine the applicability of ELR hydrogels in drug screening, the efficacy of doxorubicin was evaluated. We selected this anticancer drug as a drug model due to its extensive use in breast cancer treatment.^87^ MCF10A, MCF7, and MDA-MB-231 cells were encapsulated in ELR hydrogels and cultured for 7 days to ensure suitable cell viability in all cases. Then, cell-laden hydrogels were treated with different concentrations of doxorubicin and incubated for 48 h. In all cases, cells growing in the ELR hydrogel showed higher resistance against doxorubicin than 2D controls (Figure 5A–C). However, these differences were only statistically significant at higher doxorubicin concentrations in the case of BCCs, and in all of the concentrations tested in the case of MCF10A. Previous studies using peptide hydrogels have also shown a similar effect on drug response in cancer cells, where the efficacy of the anticancer drug in the cell-laden gel varies depending on the drug used,^60^ showing that the drug used, cell type, or peptide used influences the drug response. IC50 values were graphically determined, as they could not be calculated using GraphPad Prism 8.0 software (GraphPad Software). IC50 values obtained were higher for ELR hydrogels than 2D cultures. Indeed, MCF10A showed a 60-fold increase (0.05 μM in 2D vs 3 μM in ELR), MCF7 a 16-fold increase (0.5 μM in 2D vs 8 μM in ELR), and MDA-MB-231 a 3.3-fold increase (0.6 μM in 2D vs 2 μM in ELR). We hypothesize that these differences in drug sensitivity could be promoted by the cell–ECM and cell–cell crosstalk in the 3D ELR hydrogel environment.^56^ Therefore, these results indicate that ELR hydrogels can enhance cell resistance against doxorubicin, especially in the noninvasive cell line MCF7, providing a platform that can recreate the oncology drug response. Nevertheless, each individual BCC type should be evaluated individually, as the MDA-MB-231 invasive breast cancer cell line showed a higher sensitivity to doxorubicin, which can be associated with its higher cell proliferation and has been previously observed in other biomaterials.^56^

## 4 Conclusions

In this work, we have evaluated whether ELR polypeptides could be suitable candidates to recreate the breast cancer ECM for 3D *in vitro* cancer modeling. Hydrogels were fabricated with two ELR polypeptides, one containing cell adhesion motifs and another having MMPs-cleavage sites. Both polypeptides were functionalized with azide or cyclooctyne to enable gel formation through click chemistry and hydrophobic interactions. Nontumorigenic breast epithelial cells and BCCs were encapsulated within the hydrogels and showed optimal cell viability and proliferation for 1 week. MCF10A cells did not form acini in the gels but did form spheroids on day 7. MCF-7 formed spheroids on day 7, which increased in size on day 14, and MDA-MB-231 formed loose networks on the gels.

Cells were able to produce ECM proteins in ELR hydrogels, being collagen IV, and fibronectin was the most secreted by MCF10A and BCCs, respectively. In addition, ELR hydrogels allow the study of BCC invasiveness, as MDA-MB-231 could invade the gels after 48 h in culture and showed a higher drug resistance against doxorubicin. Therefore, these findings suggest that ELR hydrogels could be suitable for developing breast cancer models to study drug resistance and cell invasion and to evaluate the secretion of ECM by BCCs. Further studies using ELRs with different stiffnesses as well as different tumor–ECM motifs will manifest the importance of this biomaterial in deciphering the importance of the ECM in cancer progression and drug response in breast cancer.

## Supporting Information Available

Larger images of the cellular morphology in cell-laden ELR hydrogels after 1, 3, 7, 10, and 14 days of incubation and expression of ECM by MCF10A, MCF7, and MDA-MB-231 cells after 2 weeks in culture (PDF)
