# Supplementary material for: Elastin-like Recombinamer Hydrogels as Platforms for Breast Cancer Modeling
Source: Biomacromolecules. 2023 Jan 4;24(10):4408–18. doi: 10.1021/acs.biomac.2c01080 (PMC10565832; doi:10.1021/acs.biomac.2c01080)
Supplement: Supplementary file 1 — bm2c01080_si_001.pdf [file bm2c01080_si_001.pdf]

Elastin-like recombinamer hydrogels as platforms for breast cancer modeling

Barbara Blanco-Fernandez[†‡*], *Arturo Ibañez-Fonseca*[ƻ], *Doriana Orbanic*[ƻ], *Celia Ximenes-Carballo*[†], *Soledad Perez-Amodio*[†], *Jose Carlos Rodríguez-Cabello*[ƻ], *Elisabeth Engel*[¥†‡*]

[†]Institute for Bioengineering of Catalonia (IBEC), The Barcelona Institute of Science and Technology (BIST), Baldiri Reixac 10-12, Barcelona 08028, Spain; [‡] CIBER en Bioingeniería, Biomateriales y Nanomedicina, CIBER-BBN, Madrid 28029, Spain; [ƻ]BIOFORGE Lab, CIBER-BBN, University of Valladolid, Paseo de Belén 19, 47011 Valladolid, Spain; and [¥]IMEM-BRT Group, Dept. Materials Science and Engineering, EEBE, Technical University of Catalonia (UPC), Barcelona 08019, Spain; Institute for Bioengineering of Catalonia (IBEC), The Barcelona Institute of Science and Technology (BIST), Baldiri Reixac 10-12, Barcelona 08028, Spain.

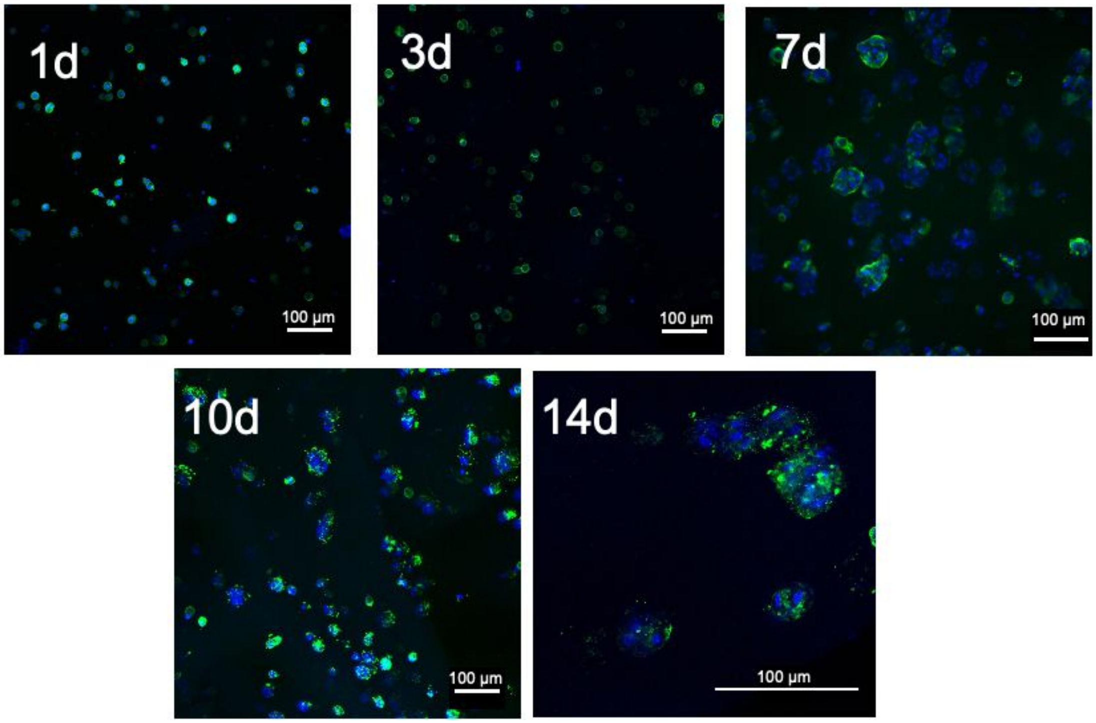

Figure S1. Cellular morphology in MCF10A-laden ELR hydrogels over time (green: cytoskeleton; blue: nuclei; scale bar = 100 μm). Images were acquired with a confocal microscope.

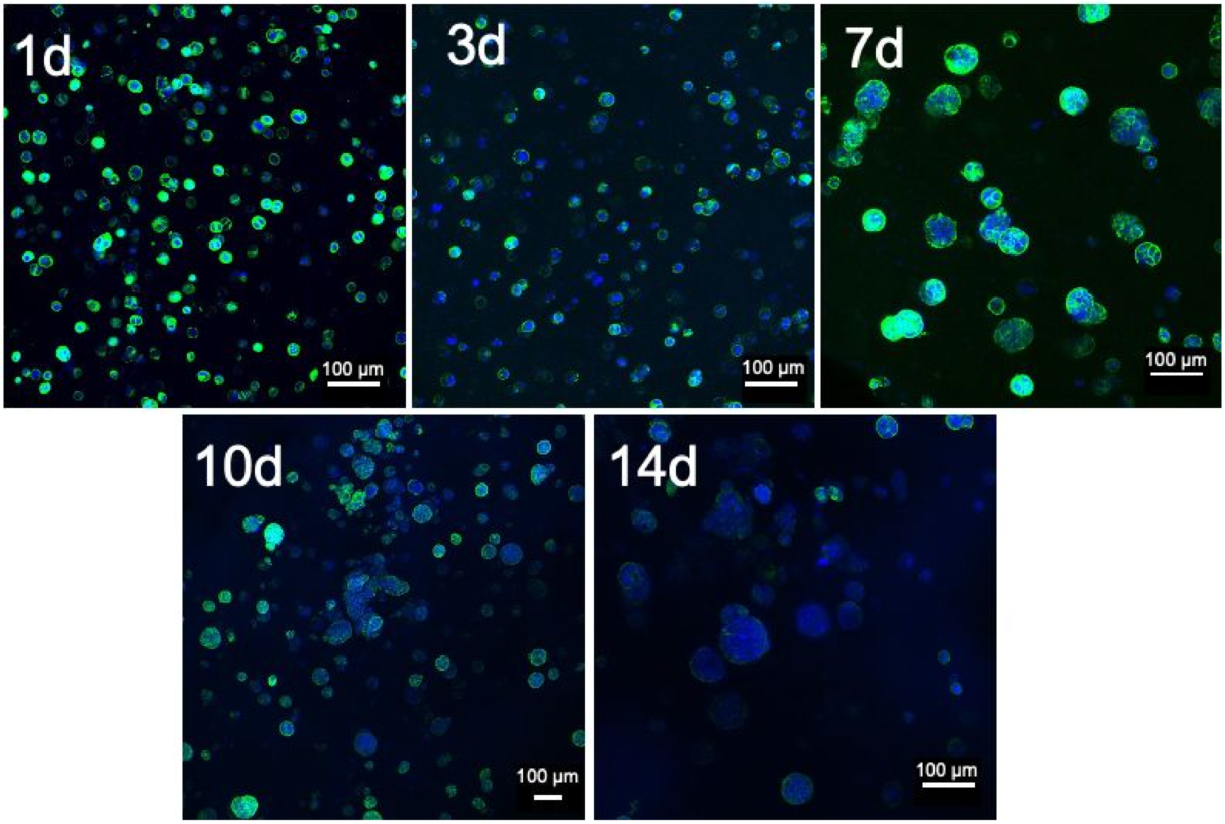

Figure S2. Cellular morphology in MCF7-laden ELR hydrogels over time (green: cytoskeleton; blue: nuclei; scale bar = 100 μm). Images were acquired with a confocal microscope.

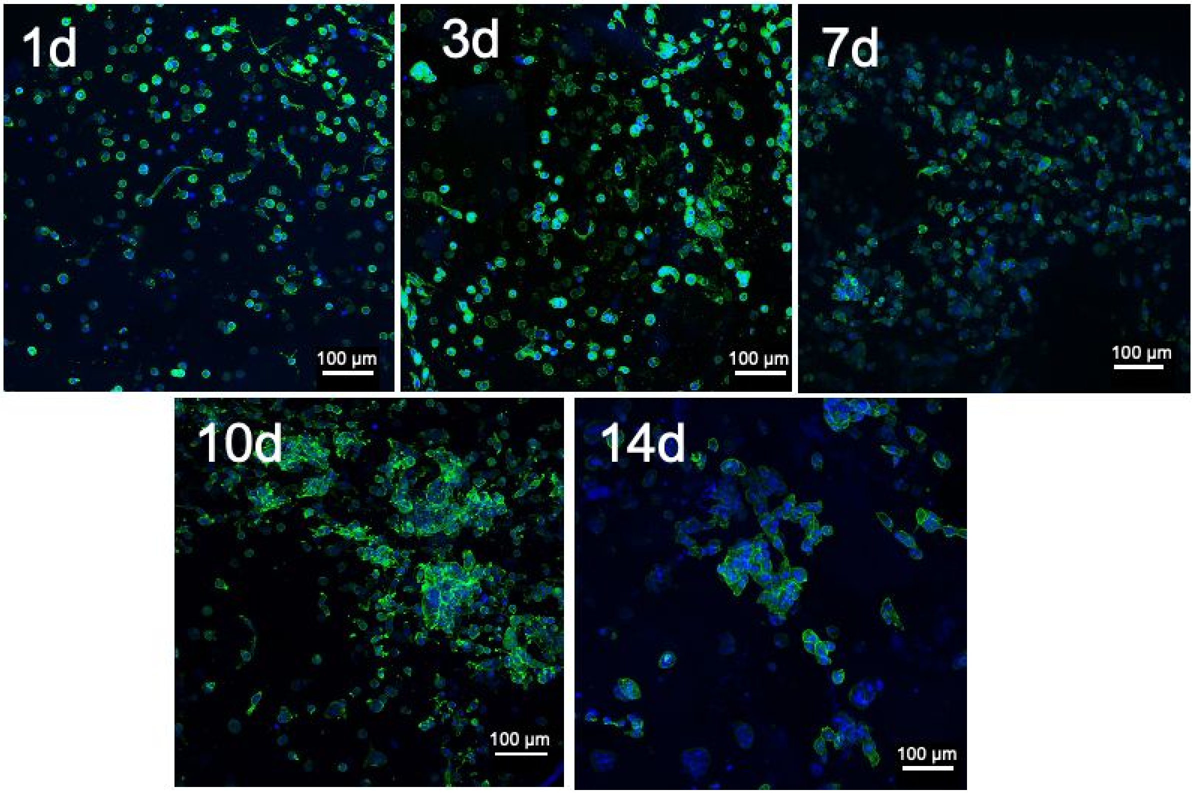

Figure S3. Cellular morphology in MDA-MB-231-laden ELR hydrogels over time (green: cytoskeleton; blue: nuclei; scale bar = 100 μm). Images were acquired with a confocal microscope.

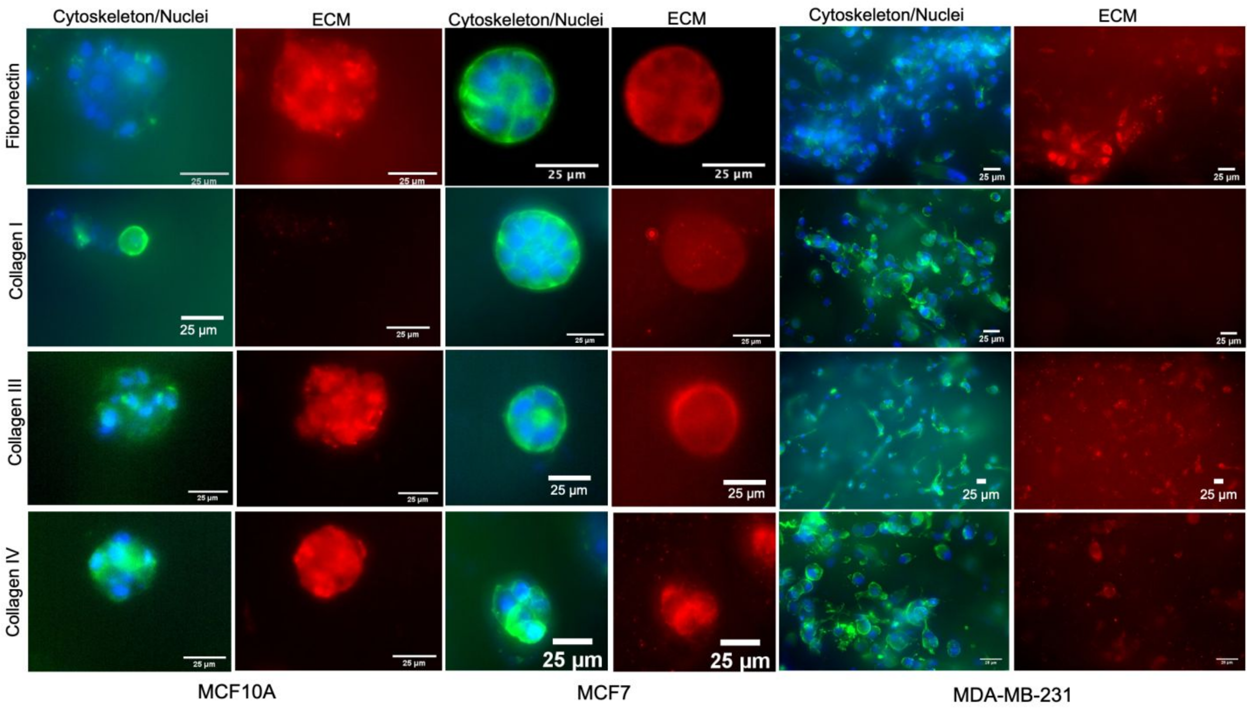

Figure S4. Production of fibronectin and collagen I, III, and IV by MCF10A, MCF7 and MDA-MB-231 cells in ELR hydrogels after 14 days. Close-ups of the spheroids stained with phalloidin-488, DAPI, and antibody against the ECM (green: cytoskeleton; blue: nuclei; red: ECM, scale bar = 25 μm).
